# Cardiac function in relation to myocardial injury in hospitalised patients with COVID-19

**DOI:** 10.1007/s12471-020-01458-2

**Published:** 2020-07-08

**Authors:** F. M. A. van den Heuvel, J. L. Vos, Y. Koop, A. P. J. van Dijk, A. L. Duijnhouwer, Q. de Mast, F. L. van de Veerdonk, F. Bosch, B. Kok, M. G. Netea, J. Hoogerwerf, W. Hoefsloot, E. T. T. L. Tjwa, C. L. de Korte, R. R. J. van Kimmenade, R. Nijveldt

**Affiliations:** 1 Department of Cardiology, Radboud University Medical Center, Nijmegen, The Netherlands; 2 Department of Internal Medicine and Radboud Center for Infectious Diseases, Radboud University Medical Center, Nijmegen, The Netherlands; 3 Department of Internal Medicine, Radboud University Medical Center, Nijmegen, The Netherlands; 4 Department of Pulmonology, Radboud University Medical Center, Nijmegen, The Netherlands; 5 Department of Gastroenterology and Hepatology, Radboud University Medical Center, Nijmegen, The Netherlands; 6 Department of Radiology, Radboud University Medical Center, Nijmegen, The Netherlands

**Keywords:** COVID-19, SARS-CoV‑2, Echocardiography, Ultrasound, Global longitudinal strain, Troponin, NT-proBNP, Myocardial injury

## Abstract

**Background:**

Previous studies have reported on myocardial injury in patients with coronavirus infectious disease 19 (COVID-19) defined as elevated cardiac biomarkers. Whether elevated biomarkers truly represent myocardial dysfunction is not known. The aim of this study was to explore the incidence of ventricular dysfunction and assess its relationship with biomarker analyses.

**Methods:**

This cross-sectional study ran from April 1 to May 12, 2020, and consisted of all consecutively admitted patients to the Radboud university medical centre nursing ward for COVID-19. Laboratory assessment included high-sensitivity Troponin T and N‑terminal pro-B-type natriuretic peptide (NT-proBNP). Echocardiographic evaluation focused on left and right ventricular systolic function and global longitudinal strain (GLS).

**Results:**

In total, 51 patients were included, with a median age of 63 years (range 51–68 years) of whom 80% was male. Troponin T was elevated (>14 ng/l) in 47%, and a clinically relevant Troponin T elevation (10 × URL) was found in three patients (6%). NT-proBNP was elevated (>300 pg/ml) in 24 patients (47%), and in four (8%) the NT-proBNP concentration was >1,000 pg/ml. Left ventricular dysfunction (ejection fraction <52% and/or GLS >−18%) was observed in 27%, while right ventricular dysfunction (TAPSE <17 mm and/or RV S’ < 10 cm/s) was seen in 10%. There was no association between elevated Troponin T or NT-proBNP and left or right ventricular dysfunction. Patients with confirmed pulmonary embolism had normal right ventricular function.

**Conclusions:**

In hospitalised patients, it seems that COVID-19 predominantly affects the respiratory system, while cardiac dysfunction occurs less often. Based on a single echocardiographic evaluation, we found no relation between elevated Troponin T or NT-proBNP, and ventricular dysfunction. Echocardiography has limited value in screening for ventricular dysfunction.

**Electronic supplementary material:**

The online version of this article (10.1007/s12471-020-01458-2) contains supplementary material, which is available to authorized users.

## What’s new

- Standard echocardiography has limited value for routine screening in hospitalised COVID-19 patients.
- We found no relation between elevated Troponin T or NT-proBNP and ventricular dysfunction.

## Introduction

The recent pandemic of coronavirus infectious disease 19 (COVID-19) has a humongous impact on global society, mainly due to the high mortality rates on the one hand and the lack of a vaccine on the other. Data presented from China and New York reported a substantial number of patients with myocardial injury in COVID-19, however this was mainly based on elevated cardiac troponin levels [1, 2]. Elevation of troponin in COVID-19 patients is associated with adverse prognosis and fatal outcome [2, 3]. In theory, causes of myocardial injury in COVID-19 are diverse, ranging from myocarditis, microangiopathy, type 1 and type 2 myocardial infarction, and myocardial injury secondary due to cytokine storm [4, 5]. There is limited data on the association between myocardial injury and the incidence of left and right ventricular dysfunction potentially caused by COVID-19. To the best of our knowledge, there are currently no recommendations about cardiac surveillance and cardiac function assessment in hospitalised COVID-19 patients. In our opinion, it is important that we recognise COVID-19 patients with cardiac dysfunction in the early stages of the disease in order to start heart failure therapy in time and to monitor these patients closely. Also, a structured follow-up after discharge is crucial to prevent cardiac remodelling and heart failure. Therefore, the aim of this study was to explore the incidence of left and right ventricular dysfunction in hospitalised patients with COVID-19, and to assess its relation to biomarker analysis.

## Methods

### Patient population

This single centre, cross-sectional study ran at the Radboud University Medical Center (Nijmegen, the Netherlands) between April 1 and May 12, 2020. We enrolled confirmed COVID-19 patients hospitalised at the COVID-19 ward who had symptoms for more than five days and who were able to provide written informed consent. Polymerase chain reaction (PCR) testing of a nasopharyngeal sample and a non-contrast-enhanced low-dose computed tomography (CT) scan of the thorax were used to confirm the COVID-19 diagnosis. COVID-19 Reporting and Data System (CO-RADS) [6] was used for standardised assessment of pulmonary involvement of COVID-19 on CT and a CT severity score was calculated [7]. There were no exclusion criteria. All patients were in comparable, stable respiratory state at the COVID-19 ward, and were haemodynamically stable without inotropic support. Patient data, including demographics, medical history, diagnostics, laboratory examinations, treatment, cardiovascular complications during hospitalisation, and outcomes were collected and analysed. The study protocol was approved by the local Medical Ethics Committee and written informed consent was obtained from all patients.

### Cardiac biomarkers

High-sensitive Troponin T and N‑terminal pro-B-type natriuretic peptide (NT-proBNP) concentrations (Roche Diagnostics, Penzberg, Germany) were measured within 48 h of the echocardiographic assessment from blood samples already taken for clinical practice. Elevation in Troponin T levels were defined as a value >14 ng/l (i.e. 99th percentile of upper reference limit [URL]). Acute myocardial injury or infarction was defined as a Troponin T rise and/or fall >20% and at least one value above 99th percentile of URL according to the fourth universal definition of myocardial infarction [8]. Elevated NT-proBNP was defined as a value >300 pg/ml.

### Echocardiographic assessment

All patients underwent a single transthoracic echocardiogram (TTE), to evaluate left and right ventricular function, diastolic left ventricular function and left ventricular global longitudinal strain (GLS). We did not extensively assess valvular function or systolic pulmonary artery pressure. All echocardiograms were performed by two experienced, EACVI TTE certified sonographers using one single ultrasound system (Affiniti70 General, Philips Healthcare, Best, the Netherlands). Offline analysis was performed by one single investigator (FvdH) using dedicated software (AGFA Enterprise Imaging Cardiology version 8.1.2, AGFA HealthCare, Mortsel, Belgium). GLS was measured using speckle tracking echocardiography on a three beats acquisition with a frame rate >60 frames/sec. All measurements were done according to the EACVI recommendations for cardiac chamber quantification [9].

Left ventricular function was evaluated by left ventricular ejection fraction (LVEF) and GLS. Preferably a triplane LVEF was used. In case of poor image quality, Simpson’s biplane LVEF was measured, or LVEF was estimated by eyeball assessment. Left ventricular dysfunction was defined as LVEF below 52% [9] and/or GLS worse than −18% [10]. If a previous echocardiography was available, left ventricular dysfunction was defined as a decrease of LVEF >5% and a value <52% and/or increase in GLS of 2% or more and a GLS worse than −18%, as compared to the previous echocardiogram.

Right ventricular dysfunction was defined as a tricuspid annular plane systolic excursion (TAPSE) < 17 mm [9] and/or a right ventricular systolic excursion velocity (RV S’) <10 cm/s [9]. If a previous echocardiography was available, right ventricular dysfunction was defined as a decrease in TAPSE >2 mm with a value <17 mm and/or a decrease in RV S’ >2 cm/s with a value <10 cm/s.

Left ventricular diastolic function was assessed by the ratio between early mitral inflow velocity and mitral annular early diastolic velocity (E/e’) and defined by an E/e’ ratio >14.

For the ultrasound examinations we collaborated with our colleagues of the department of infectious diseases, internal medicine, hepatology, and pulmonology. Patients were approached once for an ultrasound examination of the heart, lungs and liver in one single session, performed by one single sonographer. This to reduce the usage of personal protective equipment, the risk of transmission and the burden on patients.

### Statistical analysis

Categorical variables are summarised with counts and percentages, continuous variables with median and interquartile range (IQR) (non-normal distribution). All statistical analyses were performed using R for statistical computing and graphics (version 3.6.2, R Foundation, Vienna, Austria). Subgroup analyses were performed using the nonparametric Mann-Whitney U test for (unpaired) continuous variables and a Fisher’s exact test for categorical variables. The following subgroups were defined: intensive care unit (ICU) admission, pulmonary embolism, elevated Troponin T and elevated NT-proBNP. At the time of statistical analysis seven patients were still hospitalised.

## Results

### Study population

In total, 51 patients were included. The median age was 63 years (IQR; 51–68), 80% was male and the median body mass index was 27 kg/m^2^ (Tab. 1). Hypertension (41%) was the most frequent comorbidity followed by diabetes mellitus (18%) and chronic respiratory diseases (12%). Twenty-two percent of patients had a history of cardiac disease. In nine patients (17%), a previous echocardiogram was available. On admission, 22% of patients were treated with immunosuppressive therapy, 18% with angiotensin-converting enzyme inhibitors and 10% with angiotensin II receptor blockers. The most frequent symptoms at admission were fever (>38.0 °C) (94%), dyspnoea/coughing (82%), gastrointestinal complaints (29%) and chest pain (16%). In the majority of patients (92%), the diagnosis of COVID-19 was confirmed by a positive PCR test. The other 8% of the patients were considered as probable COVID-19 based on symptoms and CT findings. The median CO-RADS score was 5 and the median CT severity score was 13 (IQR; 10–16), which is considered to reflect moderate to severe respiratory involvement. Nineteen (37%) patients were admitted to the intensive care unit (ICU), of whom 17 (33%) were intubated and mechanically ventilated (median duration of mechanical ventilation 14 days [IQR; 12–20]), mostly in prone position. Three out of 19 ICU patients underwent ultrasound evaluation prior to ICU admission. The other 16 patients were first admitted to the ICU and underwent cardiac evaluation on the COVID-19 ward afterwards.

**Table 1:** Baseline characteristics of patients

	Patients (*n* = 51)
Sex, male	*n* = 41 (80%)
Age, years	63 (51–68)
Body mass index (kg/m^2^)	27 (25–29)
**Comorbidities**	
*Cardiac history*	*n* = 11 (22%)
– Obstructive coronary artery disease	*n* = 4 (8%)
– Myocardial infarction	*n* = 5 (10%)
– Non-ischaemic cardiomyopathy	*n* = 0
– Heart failure	*n* = 0
– Atrial fibrillation	*n* = 4 (8%)
– Ventricular arrythmias	*n* = 1 (2%)
– Moderate-to-severe valvular disease	*n* = 1 (2%)
– Cardiac surgery	*n* = 1 (2%)
– Cardiac electronic device	*n* = 1 (2%)
Hypertension	*n* = 21 (41%)
Diabetes mellitus	*n* = 9 (18%)
Currently smoking	*n* = 3 (6%)
Cerebrovascular disease	*n* = 2 (4%)
Chronic renal failure (GFR <30 or dialysis)	*n* = 1 (2%)
Chronic respiratory diseases (COPD/asthma)	*n* = 6 (12%)
**Diagnosis of COVID-19 infection**	
Positive PCR test	*n* = 47 (92%)
*CT scan performed*	*n* = 47 (92%)
CO-RADS classification based on the CT-scan	*n* = 44 (86%)
– CO-RADS 1–3	*n* = 2 (4%)
– CO-RADS 4	*n* = 3 (6%)
– CO-RADS 5	*n* = 26 (51%)
– CO-RADS 6	*n* = 13 (25%)
CT severity score	13 (10–16)
**Laboratory findings at admission**, median (IQR)	
Haemoglobin (mmol/l)	8.4 (7.7–9.0)
Leucocytes (10^9^/l)	7.2 (5.4–9.9)
C‑reactive protein (mg/l)	92 (45–177)
GFR (mL/min/kg/m^2^)	79 (65–90)
Troponin (median ng/l)	12 (7–21)
NT-proBNP (median pg/ml)	310 (88–565)

Values are in median and interquartile range, or n (%)

*COPD* chronic obstructive pulmonary disease, *CO-RADS* COVID-19 Reporting and Data System, *CT* computed tomography, *GFR* glomerular filtration rate, *NT-proBNP* N-terminal pro-B-type natriuretic peptide, *PCR* polymerase chain reaction

### Cardiac biomarkers

At least one Troponin T value was available in 92% of patients and two or more in 57%. Troponin T was elevated in 47% (>14 ng/l), and a Troponin T > 50 ng/l was found in six patients (12%). Three patients had a Troponin T > 140 ng/l (10 times URL), which is used as a cut-off for relevant myocardial infarction after coronary artery bypass grafting [8], with the highest Troponin T of 356 ng/l. All three needed mechanical ventilation, two had pulmonary embolism (one died), and one had an LVEF of 30%. Thirteen patients (25%) fulfilled the biochemical criteria for acute myocardial injury or infarction [8]. There was no difference in left ventricular dysfunction (*p* = 0.3) or right ventricular dysfunction (*p* = 0.6) in patients with or without criteria for acute myocardial injury or infarction.

NT-proBNP was measured in 94% of the patients. In about half of patients (47%), NT-proBNP was elevated (>300 pg/ml) and in four patients (8%) the value was >1,000 pg/ml. The maximum NT-proBNP measured was 25,000 pg/ml.

Patients hospitalised at the ICU had a higher prevalence of elevated Troponin T and a lower prevalence of elevated NT-proBNP compared with patients who had only been hospitalised at the COVID-19 ward.

### Echocardiographic findings

All echocardiographic parameters are listed in Tab. 2. Normal left and right ventricular function was observed in 69%. The median LVEF was 59.0% (IQR; 54.5–60.0%) and the median TAPSE was 22 mm (IQR; 20–27). Diastolic dysfunction was observed in only a small number of patients (*n* = 3, 6%). Only one patient had mild pericardial effusion (maximum 4 mm) without other clinical or electrocardiographic signs of pericarditis.

**Table 2:** Echocardiographic parameters of patients

	Patients (*n* = 51)
**LV dimensions and function**	
LVEDd (mm)	48 (45–52)
Indexed LVEDd (mm)	24 (22–26)
LVESD (mm)	34 (30–37)
LV mass (g/m^2^)	80 (69–89)
LVEF grading	
– LVEF ≥52%	*n* = 41 (80%)
– LVEF 46–52%	*n* = 5 (10%)
– LVEF 31–45%	*n* = 4 (8%)
– LVEF ≤30%	*n* = 1 (2%)
Global longitudinal strain (*n*)	*n* = 30 (59%)
– GLS	−18.5 (−19.7– −16.9)
– GLS ≤ −18%	*n* = 18 (35%)
– GLS > −18%	*n* = 12 (24%)
E/e’ ratio, median (IQR)	7.2 (6.1–9.2)
**RV dimension and function**	
RV basal diameter (mm)	39 (34–41) (*n* = 38)
TAPSE (mm)	22 (20–27) (*n* = 51)
RV S’ (cm)	14 (12–16) (*n* = 46)
**Atrial dimensions**	
Indexed left atrial volume (ml/m^2^)	27 (24–32)
Right atrial surface area (cm^2^)	16 (14–17)

Values are in median and interquartile range, or n (%)

*E/e’* early mitral inflow velocity/mitral annular early diastolic velocity, *EF* ejection fraction, *IQR* interquartile range, *LV* left ventricular, *LVEDd* left ventricular end-diastolic dimension, *LVESd* left ventricular end-systolic dimension, *LVEF* left ventricular ejection fraction, *LVOT* left ventricular outflow tract, *RV* right ventricular, *RV S’* right ventricular systolic excursion velocity, *TAPSE* tricuspid annular plane systolic excursion

**Fig. 1:**
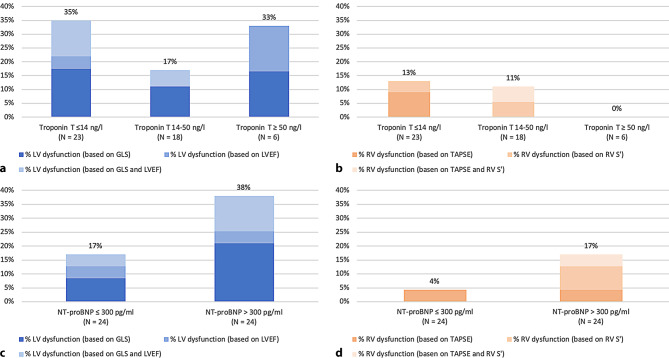
Incidence of ventricular dysfunction in relation to Troponin T and NT-proBNP. *GLS* global longitudinal strain, *LV* left ventricular, *LVEF* left ventricular ejection fraction, *NT-proBNP* N-terminal pro-B-type natriuretic peptide, *RV* right ventricular. **a** Left ventricular dysfunction in relation to Troponin T. **b** Right ventricular dysfunction in relation to Troponin T. **c** Left ventricular dysfunction in relation to NT-proBNR. **d** Reight ventricular dysfunction in relation to NT-proBNR

Fourteen patients (27%) had left ventricular dysfunction. In three patients this was due to a reduced LVEF, seven patients had abnormal GLS and in four patients both were impaired. Troponin T was elevated in five out of 14 patients with left ventricular dysfunction, of whom two had Troponin T > 50 ng/l. Elevated NT-proBNP was seen in 10 out of 14 patients with left ventricular dysfunction. Fig. 1 demonstrates the incidence of left ventricular dysfunction for each Troponin T (panel A) and NT-proBNP (panel C) category.

In five patients (10%) we observed right ventricular dysfunction. A decreased TAPSE was seen in two patients, decreased RV S’ in two patients, and both impaired in one patient. Troponin T was mildly elevated (i.e. 15 and 31 ng/l) in two out of five patients with right ventricular dysfunction, whereas an elevated NT-proBNP was found in four patients. Fig. 1 demonstrates the incidence of right ventricular dysfunction for each Troponin T (panel B) and NT-proBNP (panel D) category. One patient had an episode of decompensated heart failure, and one patient needed mechanical ventilatory support. Surprisingly, none of the patients with confirmed pulmonary embolism had right ventricular dysfunction, and right ventricular dimensions were normal.

### Outcome

The most frequent cardiovascular complications occurring during hospitalisation were pulmonary embolism (18%), atrial fibrillation (12%) and acute heart failure (6%) (see also the supplementary table). One patient died during treatment on the ICU, and five patients (10%) had acute kidney failure.

As mentioned previously, 13 patients (25%) fulfil the criteria for acute myocardial injury or infarction. In two of those patients there were symptoms and electrocardiographic signs indicating myocardial ischaemia. These two patients were eventually classified as type 2 myocardial infarction. No coronary angiography or coronary CT angiography have been performed. Also, it was impossible to further assess the reason for Troponin T rise in the other patients, due to the lack of ECG’s and reporting of chest pain symptoms.

There were no patients with a clinical suspicion of acute myocarditis, however, no further analysis (i.e. cardiac MRI or myocardial biopsy) was performed. In most patients, elevation of Troponin T was relatively low (<50 ng/l) and the LVEF was >52%, therefore, not suggestive for a clinically relevant acute myocarditis. One patient had an LVEF of 30% after a complicated COVID-19 that required mechanical ventilation and haemodialysis. In this patient Troponin T (356 ng/l) and NT-proBNP (25,000 pg/ml) were elevated and heart failure therapy was started. At the time of writing no clear cause for the left ventricular dysfunction had been found yet, but further analysis was indicated for potentially being myocarditis.

### Subgroup analyses

Subgroup analysis showed no relation between elevated Troponin T or NT-proBNP, and patients with ventricular dysfunction (see Tab. 3 and Fig. 1). Ventricular dysfunction by means of LVEF, GLS, TAPSE and RV S’ were also not significantly different between patients treated in the ICU compared with non-ICU patients. Remarkably, patients with pulmonary embolism demonstrated a higher TAPSE compared with patients without pulmonary embolism.

**Table 3:** Subgroup analysis

**Treatment**	*ICU admission* *n* *=19*	*No ICU admission* *n* *=32*	*P value*
LVEF % (median/IQR)	59.0 (55.5–60.0)	58.5 (54.4–60.0)	0.56
GLS % (median/IQR)	−19.7 (−21– −19.1)	−17.7 (−18.5– −16.4)	0.005
TAPSE mm (median/IQR)	24.0 (18.5–27.0)	22.0 (20.0–26.0)	0.3
RV S’ cm/sec (median/IQR)	15.0 (12.2–18.8)	14.0 (11.0–15.0)	0.1
Troponin T > 14 ng/l	*n* = 15 (79%)	*n* = 9 (28%)	0.005*
NT-proBNP >300 pg/ml	*n* = 4 (21%)	*n* = 20 (63%)	0.01*
**Complication**	*Pulmonary embolism* *n* *=9*	*No pulmonary embolism* *n* *=42*	*P value*
TAPSE mm (median/IQR)	27 (25–28)	21 (19–25.8)	0.008
RV S’ cm/sec (median/IQR)	16 (14–20)	14 (12–15)	0.026
Troponin T > 14 ng/l	*n* = 6 (67%)	*n* = 18 (43%)	0.46*
NT-proBNP >300 pg/ml	*n* = 3 (33%)	*n* = 21 (50%)	0.46*
**Troponin T**	*≤14* *ng/l* *n* *=23*	* >14* *ng/l* *n* *=24*	*P value*
LVEF % (median/IQR)	57 (53–60)	59 (56–60)	0.15
GLS % (median/IQR)	−18.1 (−18.7– −16.7)	−19.2 (−20– −17.1)	0.20
TAPSE mm (median/IQR)	22 (19–25.5)	22.5 (20–27)	0.44
RV S’ cm/sec (median/IQR)	12 (11–14)	15 (14–18.5)	0.004
**NT-proBNP**	*≤300* *pg/ml* *n* *=24*	*>300* *pg/ml* *n* *=24*	*P value*
LVEF % (median/IQR)	60 (57–60)	59 (54–60)	0.62
GLS % (median/IQR)	−18.8 (−19– −17.5)	−18 (−19.4– −16.5)	0.53
TAPSE mm (median/IQR)	24.5 (19–27)	21.5 (20–26.3)	0.97
RV S’ cm/sec (median/IQR)	14 (12–16.5)	14 (11.5–16.5)	0.8

All values were tested with a Mann-Whitney U test, except for (*) which was done with a Fisher’s exact test

*GLS* global longitudinal strain, *LV* left ventricular, *LVEF* left ventricular ejection fraction, *NT-proBNP* N-terminal pro-B-type natriuretic peptide*, RV S’* right ventricular systolic excursion velocity, *TAPSE* tricuspid annular plane systolic excursion

## Discussion

This study provides a unique cross-sectional observation of non-selected COVID-19 patients admitted to the ward, exploring the incidence of left and right ventricular dysfunction in relation to biomarker analyses. There was a low incidence of right ventricular dysfunction, and a relatively higher incidence of left ventricular dysfunction. However, no relation between ventricular dysfunction and elevated Troponin T nor NT-proBNP could be identified. Based on these results, Troponin T or NT-proBNP cannot be used to exclude or suggest cardiac dysfunction. Furthermore, since none of the patients with pulmonary embolism had abnormal right ventricular findings, echocardiography is not recommended as a routine screening tool to rule out pulmonary embolism.

In our study, about half of patients had elevated Troponin T. This is considerably more than described among 5,700 hospitalised COVID-19 patients in the New York City area with a prevalence in elevated troponin of 22.6% [1], and among 187 patients in Wuhan City with a prevalence of 27.8% [2]. In the latter study, elevated troponin was related to fatal outcome, and suggested to be caused by cardiac dysfunction or arrhythmia. Although we have learned that elevated Troponin T is an important prognostic marker for in-hospital mortality [11], our results indicate that this may not be directly associated with ventricular dysfunction per se. Additionally, both Troponin T and NT-proBNP in this cohort showed only mild elevation in the majority of patients, which is similar to the findings by Shi et al. where the range of biomarkers in survivors was also low [11]. They suggest that both circulating inflammatory mediators or direct viral invasion may lead to the increase in cardiac biomarkers, but also address a recent pathological study which found only limited interstitial mononuclear inflammatory infiltrates in heart tissue without myocardial damage [12]. Counter-intuitively, ICU patients had a lower prevalence of elevated NT-proBNP compared with non-ICU patients. This could potentially be caused by the small sample size, natural selection of patients who returned from the ICU with subsequent better prognosis, or inclusion of frail patients at a later stage of hospitalisation since informed consent was required, in whom NT-proBNP levels may have decreased.

There was a relatively low incidence of right ventricular dysfunction despite the relatively high incidence of acute pulmonary embolism and respiratory failure requiring mechanical ventilation. Unexpectedly, patients with pulmonary embolism had higher TAPSE, for which we could find no other explanation than the small sample size and limited severity of embolism. This study was not designed to explore the usefulness of echocardiographic screening for pulmonary embolism, though, we believe the diagnosis may be easily overlooked and patients will be undertreated when physicians depend on echocardiographic screening only. We therefore recommend CT angiography as the preferred standard for diagnosing pulmonary embolism.

Left ventricular dysfunction was not a rare observation in the present study. However, only five out of 14 patients with left ventricular dysfunction presented with elevated Troponin T and only two patients had a Troponin T concentration >50 ng/l. Also, some patients may have had chronic cardiac dysfunction, since a previous echocardiogram was only available in a limited number of patients. This emphasises that the majority of patients with elevated biomarkers had normal ventricular function. This suggests that earlier reports on myocardial injury defined as elevated biomarkers may merely reflect the severity of the disease [4], rather than the result of cardiac dysfunction or myocyte necrosis. A thoughtful interpretation of the patient’s symptoms and findings are therefore important to define the cause for biomarker elevations, and elevation in itself is not evidence for cardiac involvement. We therefore do not recommend routine echocardiography in hospitalised COVID-19 patients, also for the potential hazard for sonographers and the use of spare personal protective equipment. As the number of hospitalised COVID-19 patients is decreasing in the Netherlands [15], future studies may shift their focus from early recognition to structured follow-up to explore the prevalence of late cardiac dysfunction. Our findings confirm that COVID-19 predominantly affects the respiratory system during the acute phase, and less often the myocardium.

### General recommendations

**Fig. 2:**
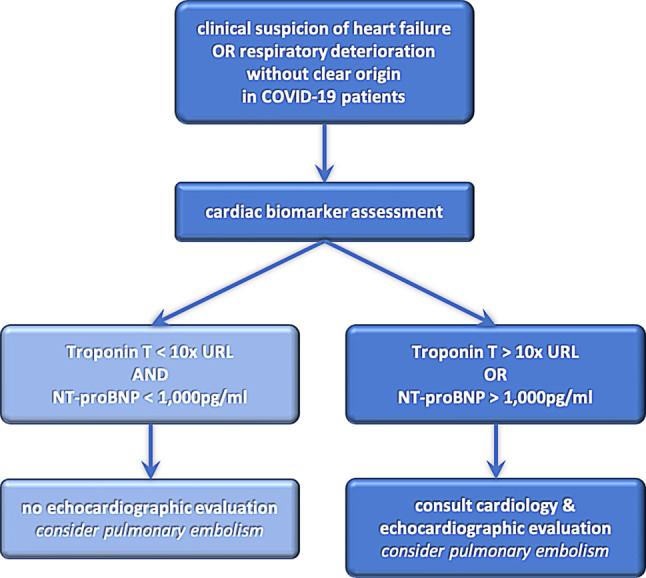
Flow chart for hospitalised, non-ICU COVID-19 patients with clinical suspicion of heart failure or respiratory deterioration

As indicated by our results, and in line with the EACVI [13], routine echocardiographic evaluation of COVID-19 patients is not recommended, and may only be considered if it is likely to change patient management. We propose to first measure cardiac biomarkers in hospitalised, non-ICU COVID-19 patients with clinical suspicion of heart failure or respiratory deterioration without a clear origin. Second, in Troponin T > 10 × URL or NT-proBNP >1,000 pg/ml we recommend further evaluation, including echocardiography and cardiology consultation (Fig. 2). It is always important to consider pulmonary embolism, which cannot be ruled out by biomarker or echocardiographic assessment. When an acute coronary syndrome is suspected, regular clinical guidelines must be followed, and not the proposed flow chart. The proposed flow chart is also supported by recent data from the Tel Aviv Medical Center [14], suggesting that the use of echocardiography should only be limited to patients with clinical deterioration.

### Limitations

Due to the explorative design of this study, some limitations need to be addressed. First, we tried to include all patients admitted to the COVID-19 ward. Since written informed consent was needed before inclusion, frail and unstable patients could not be enrolled. Secondly, to fulfil the criteria of acute myocardial injury or infarction at least two Troponin T values are necessary. In only 57% of the patients two or more Troponin T values were available. Therefore, we may have underreported the prevalence of acute myocardial injury. Finally, due to the relatively small sample size further subgroup analyses are not reliable.

## Caption Electronic Supplementary Material

**Supplementary Table.** Treatment and outcome of patients
