# Supplementary material for: Cardiac function in relation to myocardial injury in hospitalised patients with COVID-19
Source: Neth Heart J. 2020 Jul 8;28(7-8):410–7. doi: 10.1007/s12471-020-01458-2 (PMC7341471; doi:10.1007/s12471-020-01458-2)
Supplement: Supplementary file 1 — Supplementary Table. Treatment and outcome of patients [file 12471_2020_1458_MOESM1_ESM.docx]

**Supplementary Table.** Treatment and outcome of patients

|  | **Patients (*n*=47)** |
| --- | --- |
| **Treatment** |  |
| Treatment with nasal canula/face mask |  |
| Maximal given O_2_ therapy (l/min) | 6 (3-12) |
| Nasal high flow therapy | *n* = 10 (20%) |
| Maximal given O_2_ therapy (l/min) | 60 (45-60) |
| Mechanical ventilation, *n* (%) | *n* = 17 (33%) |
| Number of days | 14 (12-20) |
| Prone ventilation | *n* = 16 (31%) |
|  |  |
| Medium care unit admission | *n* = 3 (6%) |
| Intensive care unit admission | *n* = 19 (37%) |
| Days of admission | 13 (11-24) |
|  |  |
| **Complications** |  |
| Deceased | *n* = 1 (2%) |
| Acute heart failure | *n* = 3 (6%) |
| Cardiac shock requiring inotropes | *n* = 0 |
| Type 1 myocardial infarction | *n* = 0 |
| Type 2 myocardial infarction | *n* = 2 (4%) |
| Myocarditis | *n* = 0 |
| Ventricular arrythmia | *n* = 0 |
| Atrial fibrillation | *n* = 6 (12%) |
| CVA/TIA | *n* = 1 (2%) |
| Pulmonary embolism | *n* = 9 (18%) |
| Acute kidney failure | *n* = 5 (10%) |
| Septic shock requiring inotropes or vasopressors | *n* = 12 (24%) |
|  |  |
| **Hospital readmission for COVID-19** | *n* = 1 (2%) |
|  |  |
| **Discharge** |  |
| Duration of hospital admission (days) | 10 (7-20) |
| Discharge to: |  |
| Rehabilitation faculty/nursing home | *n* = 4 (8%) |
| Transfer to different hospital | *n* = 1 (2%) |
| Home (*n*, %) | *n* = 39 (76%) |

*Values are in median and interquartile range, or n (%).*

*CVA* cerebrovascular accident, *TIA* transient ischaemic attack
